# An Extremely Inhomogeneous Gross Tumor Dose is Suitable for Volumetric Modulated Arc-Based Radiosurgery with a 5-mm Leaf-Width Multileaf Collimator for Single Brain Metastasis

**DOI:** 10.7759/cureus.35467

**Published:** 2023-02-25

**Authors:** Kazuhiro Ohtakara, Kojiro Suzuki

**Affiliations:** 1 Department of Radiation Oncology, Kainan Hospital Aichi Prefectural Welfare Federation of Agricultural Cooperatives, Yatomi, JPN; 2 Department of Radiology, Aichi Medical University, Nagakute, JPN

**Keywords:** volumetric modulated arc-based radiosurgery, volumetric modulated arc therapy, stereotactic radiosurgery, multileaf collimator, dose inhomogeneity, dose gradient, dose conformity, dose distribution, brain metastasis

## Abstract

Introduction

Single or multi-fraction (mf) stereotactic radiosurgery (SRS) is an indispensable treatment option for brain metastases (BMs). The integration of volumetric modulated arc therapy (VMAT) into linac-based SRS is expected to further enhance efficacy and safety and to expand the indications for the challenging type of BMs. However, the optimal treatment design and relevant optimization method for volumetric modulated arc-based radiosurgery (VMARS) remain unestablished with substantial inter-institutional differences. Therefore, this study was conducted to determine the optimal dose distribution suitable for VMARS of BMs, especially regarding dose inhomogeneity of the gross tumor volume (GTV). The GTV boundary, not margin-added planning target volume, was regarded as a basis for planning optimization and dose prescription.

Materials and methods

This was a planning study for the clinical scenario of a single BM. Eight sphere-shaped objects with diameters of 5-40 mm in 5-mm increments were assumed as GTVs. The treatment system included a 5-mm leaf width multileaf collimator (MLC) Agility® (Elekta AB, Stockholm, Sweden) and a dedicated planning system Monaco® (Elekta AB). The prescribed dose (PD) was uniformly assigned to just cover 98% of the GTV (D_98%_). Three VMARS plans with different dose inhomogeneities of the GTV were generated for each GTV: the % isodose surfaces (IDSs) of GTV D_98%_, normalized to 100% at the maximum dose (D_max_), were ≤70% (extremely inhomogeneous dose, EIH); ≈80% (inhomogeneous dose, IH); and ≈90% (rather homogeneous dose, RH). VMARS plans were optimized using simple and similar cost functions. In particular, no dose constraint to the GTV D_max_ was assigned to the EIH plans.

Results

Intended VMARS plans fulfilling the prerequisites were generated without problems for all GTVs of ≥10 mm, whereas 86.4% was the lowest IDS for the D_98%_ for 5-mm GTV. Therefore, additional plans for 9- and 8-mm GTVs were generated, which resulted in 68.6% and 75.1% being the lowest IDSs for the D_98%_ values of 9- and 8-mm GTVs, respectively. The EIH plans were the best in terms of the following: 1) dose conformity, i.e., minimum spillage of PD outside the GTV; 2) moderate, not too excessive, dose attenuation outside the GTV, i.e., appropriate marginal dose 2-mm outside the GTV boundary as a function of GTV size; and 3) lowest dose of the surrounding normal tissue outside the GTV. In contrast, the RH plans were the worst based on all of the aforementioned measures.

Conclusions

On the assumption of uniform dose assignment to the GTV margin, a very inhomogeneous GTV dose is basically the most suitable for SRS of BMs in terms of 1) superior dose conformity; 2) minimizing the dose of the surrounding normal tissue outside the GTV; and 3) moderate dose spillage margin outside the GTV with a tumor volume-dependent rational increase, i.e., appropriate dose of the common PTV boundary. The concentrically laminated steep dose increase inside the GTV boundary for the EIH plan may also be advantageous for achieving superior tumor response, although early and excessive GTV shrinkage caused by the EIH plan during mfSRS can lead to surrounding brain injury.

## Introduction

Single- or multi-fraction (mf) stereotactic radiosurgery (SRS) has been a sine qua nontreatment option as a local radical and/or palliative therapy for brain metastases (BMs), given the current limitations and harms of open surgery, pharmacotherapy, and conventional whole brain radiotherapy (RT) [1]. The gross tumor volume (GTV) boundary has been a standard foundation for dose prescription and planning for SRS since the dawn of rigid frame fixation and even now for Leksell Gamma Knife (LGK) (Elekta AB, Stockholm, Sweden) [2,3]. However, the periphery of various margin-added planning target volumes (PTVs) has rather prevailed as a basis for dose prescription and planning in linac-based SRS (LSRS), resulting in the GTV marginal dose becoming obscure and varied, due to the considerable differences in PTV dose inhomogeneities and dose gradients inside the PTV boundary [4,5]. These current situations have made it increasingly difficult to develop a consensus regarding the optimal dose and distribution for SRS of BMs, irrespective of devices and/or techniques [5,6].

A multileaf collimator (MLC) with a 5-mm leaf width (5-mm MLC) has been commonly mounted in a general-purpose (GP) linac [7]. Volumetric modulated arc therapy (VMAT) is the most advanced and sophisticated X-ray RT technique for MLC-based linac [8-10]. Therefore, the integration of VMAT into SRS (volumetric modulated arc-based radiosurgery; VMARS), has been keenly anticipated to further enhance the efficacy, safety, and capability of LSRS [8-10]. Simultaneous and efficient irradiation of multiple BMs through a single isocenter setting is a representative praxis for VMARS [8-10]. However, the optimal dose distribution and relevant optimization method for VMARS of BMs remain unestablished, particularly regarding target dose homogeneities [4,5]. A substantial number of institutions has still emphasized a homogeneous target dose similar to 3D conformal RT, with stringent dose constraint to the D_max_ inside the GTV [11,12]. Therefore, this study was conducted to determine the optimal dose distribution suitable for VMARS using GP linac with 5-mm MLC, especially with respect to GTV dose inhomogeneity. In this study, the GTV boundary was regarded as a basis for dose prescription and planning, instead of margin-added PTV, to prioritize uniform dose prescription to the GTV boundary rather than PTV, i.e., the vast majority of the tissue at the PTV boundary being normal brain [6,13,14]. Specifically, three different dose homogeneities of GTV were compared in terms of minimization of the surrounding normal tissue doses under the same dose assignment to GTV periphery. Furthermore, the GTV dose inhomogeneity suitable for ensuring a moderate dose spillage margin, i.e., appropriate and rational dose at the commonly adopted PTV boundary, was also investigated [6,15].

This study was approved by the Clinical Research Review Board of Kainan Hospital Aichi Prefectural Welfare Federation of Agricultural Cooperatives (20220727-1). The gist of this study was previously presented at the 35th Annual Meeting of the Japanese Society for Radiation Oncology held on November 12, 2022.

## Materials and methods

**Figure 1:**
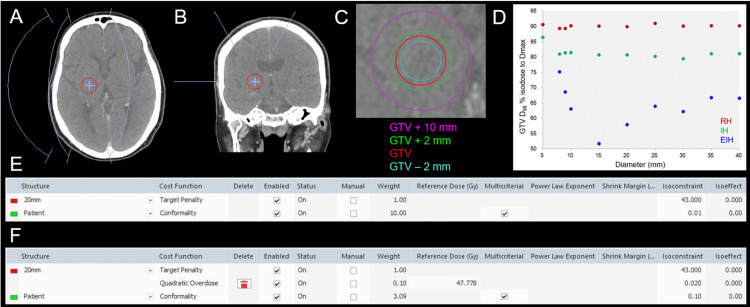
The materials and methods for this planning study and resultant dose inhomogeneities of the gross tumor volumes. The images show the location of a gross tumor volume (GTV) of 20 mm in diameter, the isocenter, and the arc arrangement (A, B); axial view (A); coronal view (B); GTV and other object volumes for evaluation (C); % isodose for GTV D_98%_ as a function of GTV diameter (D); and the cost functions (CFs) used for the optimization of volumetric modulated arc-based radiosurgery (VMARS) (E, F). (A, B) The arc arrangement consists of one coplanar arc and two non-coplanar arcs with each arc length of 120º, which are allocated to divide the cranial hemisphere evenly. The collimator angles for each arc are separately set to be 45, 90, and 135º. (D) The scatter plots show the % isodose surfaces (IDSs) covering 98% of GTV (D_98%_) normalized to 100% at the maximum dose (D_max_, D_0.001 cc_), as a function of GTV diameter, without any significant correlations (rho = -0.217, -0.467, and 0.188 for the EIH, IH and RH plans, respectively). (E) Only two cost functions (CFs) are adopted for optimization of the extremely inhomogeneous (EIH) dose plan, without any dose constraint to the D_max_ or the use of any dummy structure. The 20 mm and patient structures represent 20-mm GTV and the body surface contour, respectively. (F) Three CFs are used for the optimization of the inhomogeneous (IH) and rather homogeneous (RH) plans, in which the Quadratic Overdose CF is added to constrain the D_max_ of GTV. The VMARS optimization was simply and uniformly performed by only adjusting the isoconstraint value of the conformality CF to ensure ≥98% coverage of GTV and minimization of the surrounding tissue dose outside the GTV.

This was a planning study for the clinical scenario of a single BM. The treatment platform was a 5-mm MLC Agility® (Elekta AB, Stockholm, Sweden) mounted in a linac Infinity® (Elekta AB, Stockholm, Sweden) with a flattening filter (FF)-free mode of a 6 megavoltage X-ray beam, which provides a dose rate of up to 1400 monitor unit per minute [7,8]. The planning system Monaco® (Elekta AB, Stockholm, Sweden) was used to optimize VMARS plans [8,16]. Eight sphere-shaped objects with diameters ranging from 5 to 40 mm with a 5-mm increment were generated using a sphere drawing tool by dedicated software MIM MaestroTM (MIM Software, Cleveland, OH) and were assumed as GTVs, where the isocenters were standardized and shared as each center [6,15]. The location of the GTV, the isocenter, and the arc arrangement are shown in Figures 1A-1F.

The prescribed dose (PD) was uniformly assigned to just cover 98% of GTV (D_98%_) [13]. Three VMARS plans with different dose inhomogeneities of the GTV were generated for each GTV; the % isodose surfaces (IDSs) for GTV D_98%_, normalized to 100% at the maximum dose (D_max_), were 1) ≤70%, extremely inhomogeneous dose (EIH), 2) ≈80%, inhomogeneous dose (IH), and 3) ≈90%, rather homogeneous dose (RH). VMARS plans were optimized with the Pareto mode, using the simplest and similar cost functions (Figures 1E, 1F). In the EIH plans, the minimization of surrounding tissue dose outside the GTV was prioritized, without any dose constraint to the D_max_ of GTV. Following the completion of VMAT optimization, the doses were rescaled for PD to coincide with the GTV D_98%_.

Dose conformity to the GTV was evaluated as the spillage volume of PD outside the GTV, since various conformity indices strongly depend on the GTV [17,18]. The steepness of the dose gradient outside the prescribed IDS was evaluated using the gradient index (GI) for PD [4,19]. Normal tissue doses were compared using the spillage volumes of 75%, 50%, and 25% of PD outside the GTV. The D_98%_ of the 2-mm outside the GTV, relative to PD of 100%, was compared to evaluate the appropriateness of the dose spillage margin outside the GTV, which corresponds to the general PD to the margin-added PTV boundary for the many institutions implementing LSRS [4-6]. In addition, to evaluate the clinical implications of the D_98%_ of GTV + 2 mm, 24 Gy in single fraction (fr), 36.3 Gy in 3 fr, 43 Gy in 5 fr, 53 Gy in 10 fr, and 56.8 Gy in 13 fr were assigned to the GTV D_98%_ of possible candidate GTVs [6,15]. These dose-fractionation schemes correspond to 80-81.6 Gy of the biological effective dose (BED) based on the linear-quadratic formula with an alpha/beta ratio of 10 (BED_10_), which has usually been adopted for mfSRS in our institution since 2018, although our clinical experience with 56.8 Gy in 13 fr was preliminary and limited for selected BM cases of >25-30 cm^3^ that were deemed not amenable to 10-fr SRS [6,15,20]. Subsequently, to estimate the risk of late brain damage, the total irradiated isodose volumes receiving specific doses or those excluding GTV were compared. V12 Gy >5 cm^3^ in a single fr, 20 Gy volume >20 cm^3^ in 3 fr, and 24 Gy volume >20 cm^3^ in 5 fr are associated with an increased risk of brain radionecrosis [21,22]. As references, V40 Gy and V44.4 Gy were compared for 10 fr and 13 fr, respectively, for which 40 Gy and 44.4 Gy correspond to 120 Gy and 120.2 Gy, respectively, for BED with an alpha/beta ratio of 2 (BED_2_). The D_98%_ of the 2-mm inside the GTV, relative to PD of 100%, was also compared to evaluate the degree of dose increase inside the GTV boundary, which can affect tumor response and the adverse radiation effect (ARE) [6].

For statistical analyses, paired nonparametric tests were used, considering the distributions of the variables. Box-and-whisker plots were used to represent the distributions of variables. Friedman’s test (FT) and Scheffe’s post hoc test (SPHT) were used to compare three numerical variables. Spearman’s rank correlation coefficient (SRCC) was used to evaluate any correlations between two numerical variables. The Wilcoxon signed-rank test (WSRT) was used to compare two numerical variables. Significance was considered at P < 0.05 (*), P < 0.01 (**) and P < 0.001 (***).

## Results

**Figure 2:**
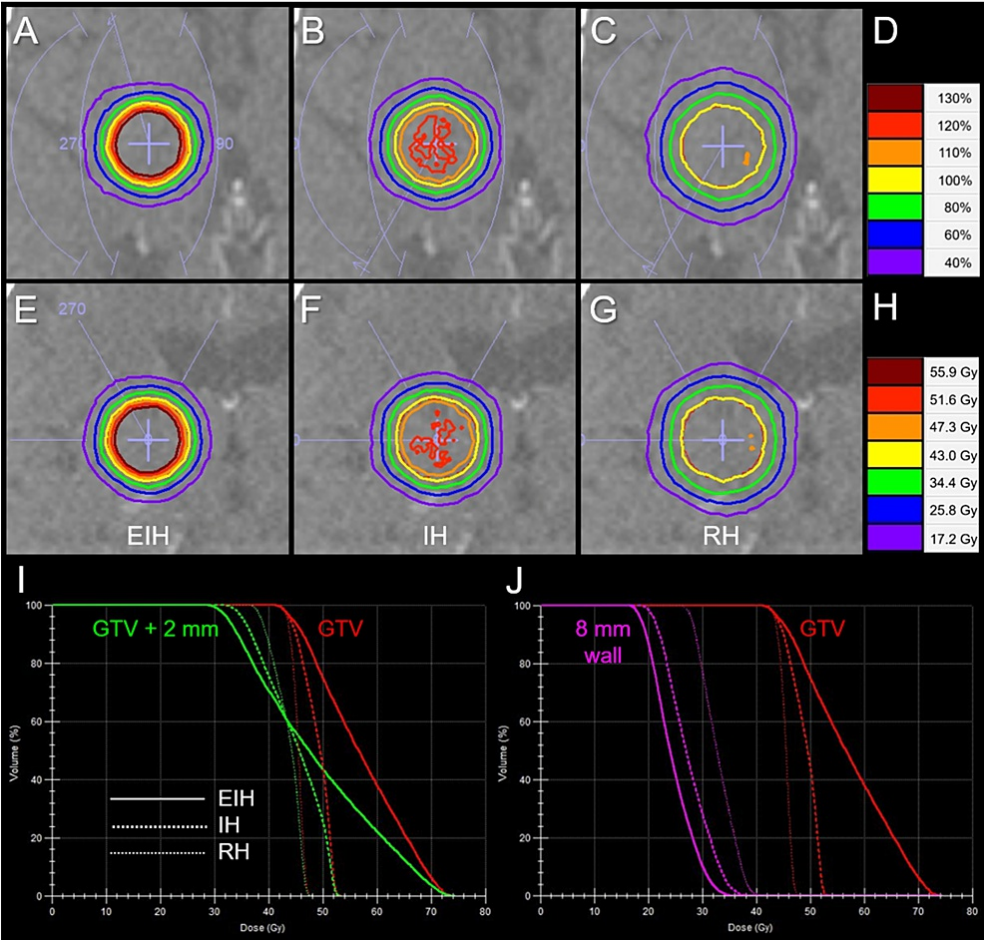
Comparison of dose distributions and dose-volume histograms for a gross tumor volume of a 20-mm-diameter tumor. The images show dose distributions (A-C, E-G); axial views (A-C); coronal views (E-G); EIH plan (A, E); IH plan (B, F); RH plan (C, G); representative % isodoses normalized to 100% at the prescribed dose (PD) (D); representative isodoses with 43 Gy in five fractions assigned to GTV D_98%_ (H); dose-volume histograms (DVHs) (I, J). (H, I)  As a clinical example, 43 Gy in five fractions is assigned to the GTV D_98%_. (I) The DVHs of GTV and GTV + 2 mm object show the differences in the dose inhomogeneities of GTV and the marginal doses of GTV + 2 mm. (J) The 8-mm wall object is defined as (GTV + 10 mm) minus (GTV + 2 mm). GTV, gross tumor volume; EIH, extremely inhomogeneous; IH, inhomogeneous; RH, rather homogeneous

Three VMARS plans fulfilling the intended prerequisites were generated without problems for all GTVs ≥10 mm. The EIH plan for a 15-mm GTV resulted in the most inhomogeneous GTV dose (Figure 1D). For a 5-mm GTV, 86.4% was the lowest IDS for the GTV D_98%_, although the RH plan was generated (Figure 1D). Therefore, additional plans for 9- and 8-mm GTVs were generated, which resulted in 68.6% and 75.1% (>70%) being the lowest IDS values for the GTV D_98%_, respectively. IH and RH plans were able to be generated for 9- and 8-mm GTVs. The % IDSs of GTV D_98%_ for the most inhomogeneous GTV doses significantly decreased as a function of GTV diameter ranging from 5 mm to 15 mm (SRCC, P < 0.001 ***, rho = -1.000), while they increased for GTV diameter ranging from 15 mm to 40 mm (SRCC, P = 0.019 *, rho = 0.886). Thus, the EIH plans with the GTV D_98%_ ≤70% IDS were achieved for GTV ≥9 mm. Therefore, subsequent comparisons of three plans with different GTV dose inhomogeneities were limited to 21 plans for GTV ≥10 mm. Comparisons of dose distributions and dose-volume histograms (DVH) for three plans for 20-mm GTV are shown in Figures 2A-2J, in which the marginal dose of GTV + 2 mm and the surrounding normal tissue doses were the lowest for the EIH plan.

**Figure 3:**
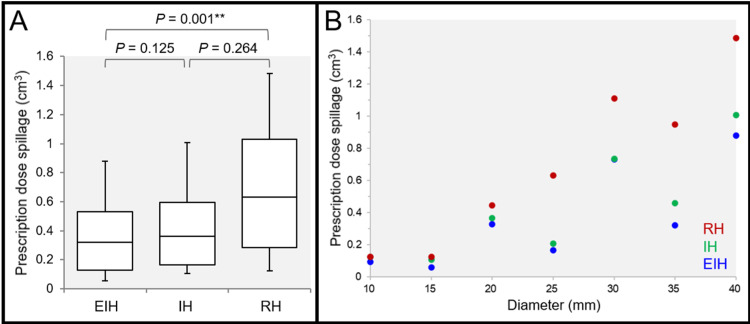
Comparison of the spillage volume of the prescribed dose outside the gross tumor volume of a tumor ≥10 mm in diameter. The images show box-and whisker plots (BWP) along with the results of Friedman’s test (FT) and Scheffe’s post hoc test (SPHT) (A); and the scatter plots for the spillage volume of the prescribed dose (PD) outside the GTV as a function of GTV diameter. (A, B) The spillage volume of the PD outside the GTV was defined as the irradiated isodose volume (IIV) of the PD minus 98% volume of the GTV. (A) FT demonstrated a significant difference among the three groups (P = 0.001 **, Kendall’s coefficient of concordance [KCC] = 0.968). (B) The spillage volumes of the PD were significantly correlated with the GTV diameter in the EIH plan (P = 0.036 *, rho = 0.786), IH plan (P = 0.007 **, rho = 0.893), and RH plan (P < 0.001 ***, rho = 0.964) GTV, gross tumor volume; EIH, extremely inhomogeneous; IH, inhomogeneous; RH, rather homogeneous

The spillage volumes of PD outside the GTV were significantly different among the three groups: lowest in the EIH plans, and highest in the RH plans (Figures 3A, 3B).

**Figure 4:**
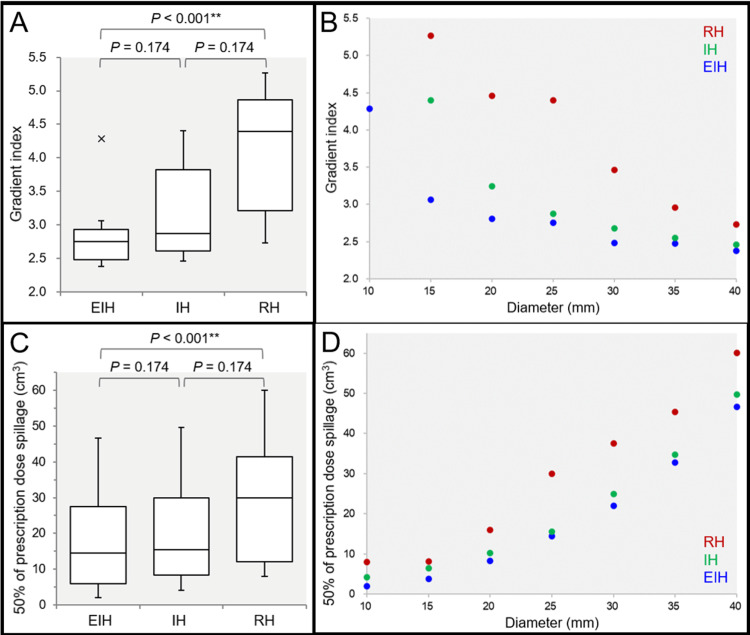
Comparison of the gradient index and the spillage volume of 50% of the prescribed dose outside the gross tumor volume of ≥10 mm. The images show the gradient index (GI) (A, B); the spillage volume of 50% of the PD outside the GTV (C, D); BWPs along with the results of FT and SPHT (A, C); and the scatter plots (B, D). (A, B) The GI is defined as the ratio of the IIV for 50% of the PD to that for 100% of the PD. (C, D) The spillage volume of 50% of the PD outside the GTV was defined as the IIV of 50% of the PD minus the GTV. (A) The x-indication beyond the whisker shows an outlier >1.5 times the interquartile range (IQR). (A, B) Two outliers of GIs in the IH and RH plans for the 10-mm GTV, 7.57 and 13.78, respectively, are not shown. (A, C) FT proved the significant differences among the three groups for the GI (P < 0.001 ***, KCC = 1.000) and the spillage volume (P < 0.001 ***, KCC = 1.000). (B, D) The scatter plots show the GI (B) and the spillage volume of 50% of the PD (D) as a function of GTV diameter, with significant correlations (rho = -1.000 and 1.000, respectively). EIH, extremely inhomogeneous; IH, inhomogeneous; RH, rather homogeneous; BWP, box-and whisker plot; FT, Friedman’s test; SPHT, Scheffe’s post hoc test; PD, prescribed dose; GTV, gross tumor volume; IIV, irradiated isodose volume; KCC, Kendall’s coefficient of concordance

The spillage volumes of PD increased significantly as a function of GTV diameter, in which the degree of increment was lowest in the EIH plans and highest in the RH plans. The steepness of dose falloff outside the prescribed IDS was significantly different among the three groups: steepest in the EIH plans and most gradual in the RH plans (Figures 4A-4D).

The strong inverse correlation of GI with GTV size was also revalidated (Figure 4B) (JRR). The spillage volumes of 50% of PD outside the GTV were significantly different among the three groups: lowest in the EIH plans and highest in the RH plans (Figures 4C, 4D). The 50% spillage volumes for the EIH plans were significantly lower than those for the IH plans on WSRT (P = 0.018 *), whereas they were not significantly different between the two groups on SPHT (Figure 4C). The 50% spillage volumes increased significantly as a function of GTV diameter, irrespective of the GTV dose inhomogeneities (Figure 4D). The spillage volumes of 75% of PD outside the GTV were also significantly different among the three groups: lowest in the EIH plans and highest in the RH plans (data not shown). More specifically, FT demonstrated the significant difference in the 75% spillage volumes (P < 0.001 ***), and SPHT showed a significant difference between the EIH and RH plans (P < 0.001 ***). Furthermore, the spillage volumes of 25% of PD outside the GTV were also significantly different among the three groups (data not shown): FT demonstrated a significant difference in the 25% spillage volumes (P = 0.005 **), and SPHT showed significant differences between the EIH and RH plans (P = 0.014 *) and between the IH and RH plans (P = 0.028 *). However, there was no significant difference between the EIH and IH plans (WSRT, P = 0.398).

**Figure 5:**
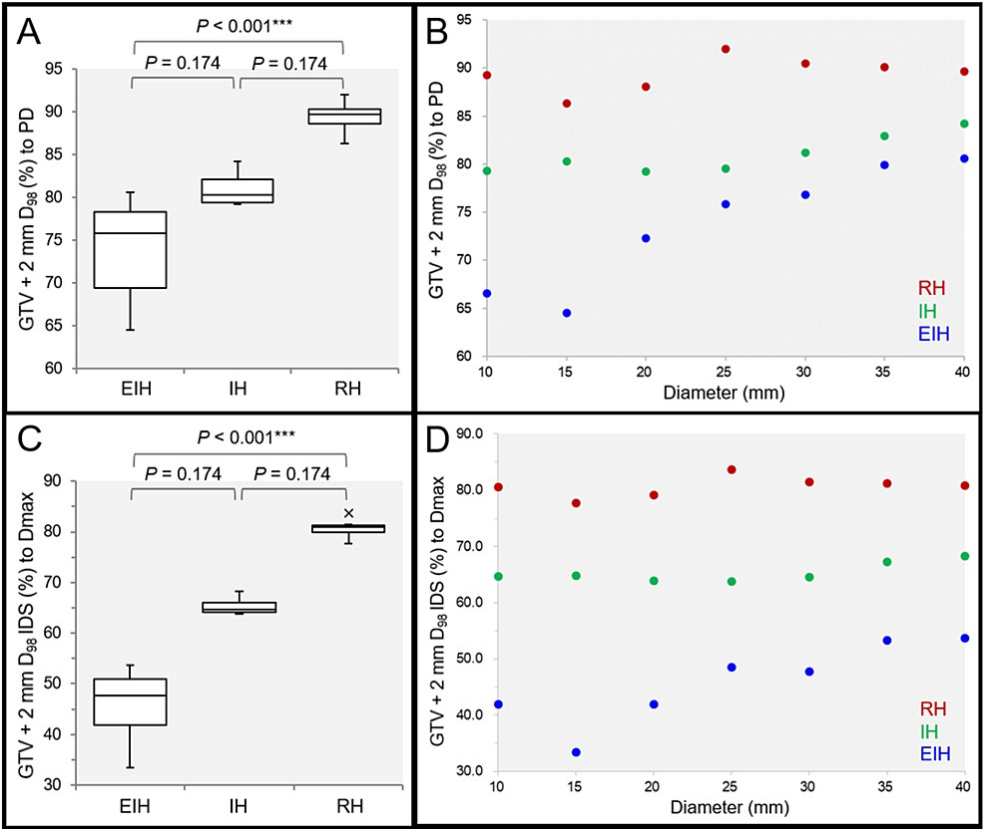
Comparison of the marginal doses of 2 mm outside the gross tumor volume. The images show the GTV + 2 mm D_98%_ (%) relative to 100% of the PD (A, B); the the GTV + 2 mm D_98%_ IDS (%) relative to 100% of the D_max_ (C, D); the BWPs along with the results of FT and SPHT (A, C); and the scatter plots (B, D). (A) FT proved significant difference among the three groups (P < 0.001 ***, KCC = 1.000). (B) The GTV + 2 mm D_98%_ relative to the PD are significantly correlated with the GTV diameter in the EIH plan (P < 0.001 ***, rho = 0.964) and IH plan (P = 0.023 *, rho = 0.821), while not in the RH plan (P = 0.215, rho = 0.536). (C) FT proved significant difference among the three groups (P < 0.001 ***, KCC = 1.000). (D) The GTV + 2 mm D_98%_ relative to the D_max_ are significantly correlated with the GTV diameter in the EIH plan (P = 0.014 *, rho = 0.851), while not in the IH plan (P = 0.337, rho = 0.429) and RH plan (P = 0.215, rho = 0.536). EIH, extremely inhomogeneous; IH, inhomogeneous; RH, rather homogeneous; BWP, box-and-whisker plot; FT, Friedman’s test; SPHT, Scheffe’s post hoc test; PD, prescribed dose; GTV, gross tumor volume; IDS, isodose surface; Dmax, maximum dose; KCC, Kendall’s coefficient of concordance

The D_98%_ doses of the 2 mm outside the GTV were significantly different among the three groups: lowest in the EIH plans and highest in the RH plans (Figures 5A-5D).

The median value of the GTV + 2 mm D_98%_ relative to the D_max_ in the EIH plan was 47.7% (<50%), being the lowest with 33.4% IDS for 15-mm GTV (Figures 5B, 5D). The GTV + 2 mm D_98%_ relative to PD in the EIH and IH plans and those relative to the D_max_ in the EIH plans increased significantly as a function of GTV diameter. When several doses corresponding to BED_10_ of 80-81.6 Gy were assigned to GTV D_98%_, the BED_10_ of the GTV + 2 mm D_98%_ ranged from approximately 40 to 60 Gy and increased as a function of GTV diameter in the EIH plans (Table 1).

**Table 1:** Comparison of the marginal doses of 2 mm outside the gross tumor volume and the surrounding normal tissue doses under dose prescriptions with the possible candidates. (*) The volumes are computed from the DVH, which are slightly different from the calculated ones, i.e., 4.16 cm^3^ versus 4.19 cm^3^. The biological effective doses based on the linear-quadratic formula with an alpha/beta ratio of 10 (BED_10_) are listed under the corresponding absolute doses in various fractions (fr). The V_X Gy_ is defined as the volume receiving ≥X Gy, outside the GTV. The Y Gy volume is defined as the total irradiated volume receiving ≥Y Gy, including the GTV. (^#^)  V_12 Gy_ in single fr >5 cm^3^. 20 Gy volume in 3 fr or 24 Gy volume in 5 fr >20 cm^3^. DVH, dose-volume histogram; EIH, extremely inhomogeneous; IH, inhomogeneous; RH, rather homogeneous

GTV	GTV D_98%_	EIH	IH	RH	EIH	IH	RH
Diameter	Dose / fr	GTV + 2 mm D_98%_			Normal tissue doses or irradiated isodose volumes		
(Volume*)	(BED_10_)	(BED_10_)					
10 mm	24 Gy/1 fr	16.0 Gy	19.0 Gy	21.4 Gy	V12 Gy		
(0.50 cm^3^)	(81.6 Gy)	(41.6 Gy)	(55.1 Gy)	(67.2 Gy)	2.02 cm^3^	4.17 cm^3^	8.01 cm^3^^#^
15 mm	24 Gy/1 fr	15.5 Gy	19.3 Gy	20.7 Gy	V12 Gy		
(1.79 cm^3^)	(81.6 Gy)	(39.5 Gy)	(56.6 Gy)	(63.6 Gy)	3.76 cm^3^	6.39 cm^3 #^	8.10 cm^3 #^
20 mm	36.3 Gy/3 fr	26.2 Gy	28.7 Gy	31.9 Gy	20 Gy volume		
(4.16 cm^3^)	(80.2 Gy)	(49.1 Gy)	(56.2 Gy)	(65.8 Gy)	10.82 cm^3^	12.66 cm^3^	18.40 cm^3^
20 mm	43 Gy/5 fr	31.1 Gy	34.1 Gy	37.9 Gy	24 Gy volume		
(4.16 cm^3^)	(80.0 Gy)	(50.4 Gy)	(57.4 Gy)	(66.6 Gy)	10.65 cm^3^	12.43 cm^3^	18.00 cm^3^
25 mm	36.3 Gy/3 fr	27.5 Gy	28.9 Gy	33.4 Gy	20 Gy volume		
(8.21 cm^3^)	(80.2 Gy)	(52.7 Gy)	(56.7 Gy)	(70.6 Gy)	19.91 cm^3^	20.95 cm^3 #^	33.58 cm^3 #^
25 mm	43 Gy/5 fr	32.6 Gy	34.2 Gy	39.5 Gy	24 Gy volume		
(8.21 cm^3^)	(80.0 Gy)	(53.9 Gy)	(57.6 Gy)	(70.7 Gy)	19.56 cm^3^	20.62 cm^3 #^	32.97 cm^3 #^
30 mm	53 Gy/10 fr	40.7 Gy	43.0 Gy	47.9 Gy	V40 Gy		
(14.08 cm^3^)	(81.1 Gy)	(57.3 Gy)	(61.5 Gy)	(70.8 Gy)	7.38 cm^3^	8.99 cm^3^	15.65 cm^3^
35 mm	53 Gy/10 fr	42.3 Gy	44.0 Gy	47.7 Gy	V40 Gy		
(22.47 cm^3^)	(81.1 Gy)	(60.2 Gy)	(63.4 Gy)	(70.5 Gy)	10.78 cm^3^	12.30 cm^3^	18.53 cm^3^
40 mm	56.8 Gy / 13 fr	45.8 Gy	47.8 Gy	50.9 Gy	V44.4 Gy		
(33.42 cm^3^)	(81.6 Gy)	(61.9 Gy)	(65.4 Gy)	(70.8 Gy)	13.18 cm^3^	15.64 cm^3^	21.46 cm^3^

However, the GTV + 2 mm D_98%_ values for the IH plans and, in particular, RH plans were generally too high for the marginal doses of the 2 mm outside the GTV boundary. In addition, normal tissue doses outside the GTV increased unfavorably as the GTV dose homogeneities increased: V_12 Gy_ in a single fr for the RH plan of 10-mm >5 cm^3^; 20 Gy volumes in 3 fr for the IH and RH plans of 25-mm >20 cm^3^; 24 Gy volumes in 5 fr for the IH and RH plans of 25-mm >20 cm^3^; and the 20 and 24 Gy volumes in the RH plans for 25-mm exceeded >10 cm^3^ compared to those in the IH plans.

**Figure 6:**
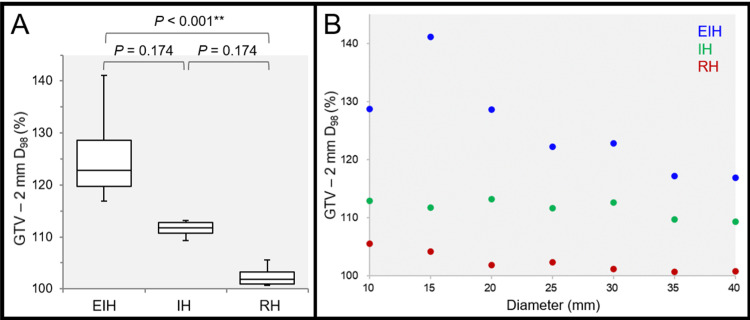
Comparison of the marginal doses of 2 mm inside the gross tumor volume. The images show the GTV - 2 mm D_98%_ (%) relative to 100% of the PD (A, B); the BWPs along with the results of FT and SPHT (A); and the scatter plots (B). (A) FT proved a significant difference among the three groups (P < 0.001 ***, KCC = 1.000). (B) The GTV - 2 mm D_98%_ values are significantly correlated with the GTV diameter in the EIH plan (P = 0.003 **, rho = -0.929), RH plan (P = 0.003 **, rho = -0.929), while not in the IH plan (P = 0.052, rho = -0.750). EIH, extremely inhomogeneous; IH, inhomogeneous; RH, rather homogeneous; BWP, box-and-whisker plot; FT, Friedman’s test; SPHT, Scheffe’s post hoc test; PD, prescribed dose; GTV, gross tumor volume; KCC, Kendall’s coefficient of concordance

Not surprisingly, the D_98%_ doses of the 2 mm inside the GTV were significantly different among the three groups: highest in the EIH plans and lowest in the RH plans, as expected (Figures 6A, 6B).

The GTV - 2 mm D_98%_ was highest in the EIH plan for 15-mm GTV (Figure 6B). It should be noted that the GTV - 2 mm D_98%_ decreased significantly as a function of GTV diameter in the EIH plans, especially for GTV ≥15 mm (Figure 6B).

**Figure 7:**
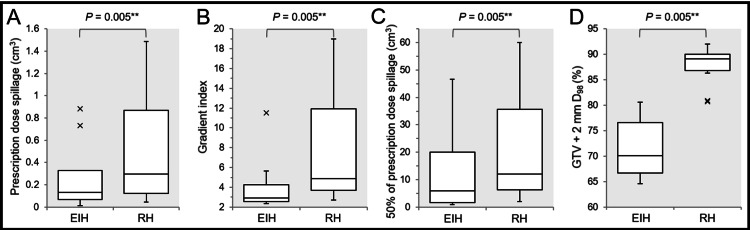
Comparison of extremely inhomogeneous versus rather homogeneous doses of gross tumor volume of 5-40 mm. The images show the BWPs along with the results of Wilcoxon signed-rank test (A-D); the spillage volume of the PD outside the GTV (A); the GI (B); the spillage volume of 50% of the PD outside the GTV (C); and the D_98%_ (%) of the GTV + 2 mm object, relative to 100% of the PD (D). (A-D) The EIH plans in this comparison include 86.4%, 75.1% and 68.6% IDS-covered plans for 5-, 8- and 9-mm GTV, respectively, in addition to the EIH plans for GTV of ≥10 mm. (A, B, D) The x-indications beyond the whiskers show outliers >1.5 times the IQR. EIH, extremely inhomogeneous; RH, rather homogeneous; GTV, gross tumor volume; BWP, box-and-whisker plot; PD, prescribed dose; IDS, isodose surface; IQR, interquartile range

Finally, a total of 10 EIH plans including 5- and 8-mm GTVs with 86.4% and 75.1% IDS covering, respectively, were compared to the corresponding EH plans (Figures 7A-7D).

The EIH plans were significantly superior to the RH plans in terms of lower spillage volume of PD outside the GTV (Figure 7A), dose gradient (Figure 7B), lower spillage volume of 50% of PD (Figure 7C), or lower and moderate marginal doses of 2 mm inside the GTV boundary (Figure 7D).

## Discussion

This planning study explored the optimal dose distribution for LSRS with VMAT for a single BM, primarily based on a different perspective from those of the previous studies and common clinical practices. That is, the present study prioritized GTV margin as a basis for dose prescription and planning and also simultaneously attempted to obtain a moderate dose spillage margin, i.e., appropriate PTV marginal dose, as dose prescription to the PTV periphery with a varied margin usually leads to inconsistent and variable GTV marginal doses [6]. In the present study, the relation of D_98%_ between the GTV and 2-mm outside was substantially varied and affected by tumor volume and planning policy.

This comparative study confirmed that affirmative allowance of excessive dose increases inside the target boundary, the EIH target dose generally leads to more precipitous dose fall-off outside the target boundary, namely a steep dose gradient, along with minimum spillage of PD, i.e., superior dose conformity. This distinctive dose distribution feature has been an essential element of SRS. A steep dose gradient is basically beneficial for normal tissue-sparing, and a concentrically laminated steep dose increase inside the target boundary likely affects clinical efficacy beyond the prescribed marginal dose. Recently, the physical and clinical advantages of an inhomogeneous target dose covered by ≤70% IDS have been increasingly acknowledged and proactively adopted in not only LGK but also LSRS [23-25].

Too steep of a dose gradient outside the GTV can impair adequate coverage of the relevant uncertainties, including microscopic brain invasion and potential tumor displacement during mfSRS [6,26-29]. Therefore, a moderate and appropriate dose attenuation margin outside the GTV boundary that is tailored to each clinical condition is also an important element for dose distribution. In the present study, when BED_10_ of 80-81.6 Gy was assigned to the GTV D_98%_, the marginal doses 2 mm outside the GTV in the EIH plans approximated the commonly adopted PD to PTV margins, such as 27 Gy in 3 fr and 30 Gy in 5 fr, whereas those of the IH and RH plans were quite excessive. Furthermore, the gradual increase of the GTV + 2 mm D_98%_ in the EIH as a function of GTV size seems to be rational in terms of ensuring higher doses for larger tumors to maintain anti-tumor efficacy. Therefore, the dose spillage margin in the EIH plans was deemed the most moderate of the three plans.

Meanwhile, potential detriments of EIH GTV dose include early and excessive GTV shrinking during mfSRS and the application to GTV with excessive exudation of contrast media, e.g., the comet-tail sign [6,30]. Early tumor response can lead to early alleviation of relevant neurological symptoms, but also gradually to unintended high-dose exposure to the surrounding normal brain [6,28,29]. In a case with an enhancing lesion exceedingly larger than the visible mass on T2-weighted images, the EIH plan likely leads to high-dose exposure to the surrounding brain outside the true GTV. A larger number of fractions is generally preferred for larger tumors to reduce the risk of ARE, and consequently, longer treatment duration renders a large BM more susceptible to tumor change and/or deviation during mfSRS [6,15,22,28,29]. However, the doses at 2 mm inside the GTV boundary in the EIH plans tended to decrease as a function of GTV size, which may mitigate the increased risk of ARE attributed to significant tumor shrinkage during mfSRS [6].

Taken together, not only single IDS for PD along with D_max_ but the balance of GTV marginal dose and the doses at approximately 2 mm outside and inside the GTV boundary, namely three-tiered dose gradient optimization and evaluation should be considered to objectively compare current varied dose distributions in depth. We have used BED_10_-based consistent dose prescription to the GTV margin along with dose gradient optimization outside and inside the GTV using modified dynamic conformal arcs and CyberKnife® with Iris® variable collimator (Sunnyvale, CA: Accuray Inc.) since 2018, for which ≤80% IDS has been used for GTV coverage [6,15]. This study also supported the application of EIH GTV dose to VMARS for BM. We have applied mf-VMRS with EIH GTV dose and BED_10_ of ≥80 Gy assigned to the GTV margin for BM since 2021.

This study has several inherent limitations. PD was uniformly assigned to GTV D_98%_ in this study. However, the <2% volume of GTV, which is part of the GTV receiving the dose under PD, increases as a function of GTV diameter, and the D_2%_ for 40-mm GTV reaches 0.67 cm^3^, which is larger than a 10-mm tumor. In practice, higher GTV coverage of >98% with PD may be preferred to mitigate the possible impairment of local control for a large BM. In addition, the dose distribution of VMARS would be affected by the differences in leaf width and/or design of the MLC, X-ray energy with or without FF, the treatment planning system, and the optimization algorithm [8-10].

The present results and arguments warrant further investigation. This study assumed a single BM with a specific location and adopted an extremely simple combination of CFs for VMARS optimization. The effect of a different optimization approach with other CFs including Target EUD (equivalent uniform dose) and different combinations of CFs on dose distribution should be examined [8,16]. In the present study, the EIH plan for 15-mm GTV showed the most inhomogeneous GTV dose with a rather too steep dose falloff outside the GTV, for which moderate dose constraint to Dmax may be necessary. A comparison of the EIH plans and inhomogeneous dose plans covered by ≈70% IDS remains unresolved. Furthermore, for simultaneous irradiation of multiple BMs, dose distribution would be substantially affected by dose interference even if the same optimization is applied. The degree of dose interference would vary according to the tumor number and its proximity.

## Conclusions

For VMARS with 5-mm MLC, a very inhomogeneous GTV dose with dose assignment to the GTV margin is essentially suitable for SRS of a single BM in terms of superior dose conformity, minimizing the dose of the surrounding normal tissue outside the GTV, and moderate dose spillage margin outside the GTV with a rational and gradual increase as a function of GTV size. Furthermore, the concentrically laminated steep dose increase inside the GTV boundary achieved by VMARS may also be beneficial for ensuring superior tumor response.
